# Anti-Apoptotic and Antioxidant Effects of 3-*Epi*-Iso-*Seco*-Tanapartholide Isolated from *Artemisia argyi* against Iodixanol-Induced Kidney Epithelial Cell Death

**DOI:** 10.3390/biom10060867

**Published:** 2020-06-05

**Authors:** Dahae Lee, Kem Ok Kim, Dongho Lee, Ki Sung Kang

**Affiliations:** 1 College of Korean Medicine, Gachon University, Seongnam 13120, Korea; pjsldh@naver.com; 2 Department of Biosystems and Biotechnology, College of Life Science and Biotechnology, Korea University, Seoul 02841, Korea; kikeko520@gmail.com

**Keywords:** apoptosis, contrast agent, cytotoxicity, iodixanol, oxidative stress

## Abstract

Iodixanol is a non-ionic iso-osmolar contrast agent, but it is a risk factor for kidney damage and increases morbidity and mortality. In this study, we investigated the effect of 9 sesquiterpenes isolated from mugwort (*Artemisia argyi*) in contrast agent-induced cytotoxicity in LLC-PK1 cells. Cells were exposed to nine sesquiterpene compounds for 2 h, followed by incubation with iodixanol for 3 h. Cell viability was assessed using the Ez-Cytox assay. The level of reactive oxygen species was measured using 2′,7′-dichlorodihydrofluorescein diacetate staining. Apoptotic cell death was detected using annexin V/PI staining. In addition, immunofluorescence staining and western blotting were performed using antibodies against proteins related to apoptosis, oxidative stress, and MAPK pathways. The most effective 3-*epi*-iso-*seco*-tanapartholide (compound **8**) among the 9 sesquiterpene compounds protected LLC-PK1 cells from iodixanol-induced cytotoxicity, oxidative stress, and apoptotic cell death. Pretreatment with compound **8** reversed iodixanol-induced increases in the expression of JNK, ERK, p38, Bax, caspase-3, and caspase-9. It also reversed the iodixanol-induced decrease in Bcl-2 expression. Furthermore, pretreatment with compound **8** caused nuclear translocation of Nrf2 and upregulated HO-1 via the Nrf2 pathway in iodixanol-treated LLC-PK1 cells. Thus, we demonstrated here that compound **8** isolated from *A. argyi* has the potential to effectively prevent iodixanol-induced kidney epithelial cell death via the caspase-3/MAPK pathways and HO-1 via the Nrf2 pathway.

## 1. Introduction

Contrast agents enhance radiodensity in a target tissue or structure. Although contrast agents are not considered harmful to the body, some side effects of contrast agents used during imaging-based investigations for the diagnosis of disease are a problem [1,2]. Contrast agent-induced nephropathy is the third most common cause of hospital acquired acute renal failure with increased mortality [3]. According to osmolality, various contrast agents are primarily divided into three groups: high and low osmolality contrast agents and iso-osmolar contrast agents [4]. Iodixanol is known as an iso-osmolar contrast agent and is widely used in the contemporary clinical setting. It is reported to have a lower risk of nephropathy compared with other contrast agents [5]. Thus, adverse reactions caused by osmolality have been reduced; however, hypersensitivity and other specific reactions induced by contrast agents are increasing [6]. Although little is known as yet about the pathogenesis of contrast-induced nephropathy, hypoxia, renal ischemia, and cytotoxic effects on renal tubular cells have been reported to cause contrast-induced nephropathy [7]. There have been few preventive strategies of contrast-induced nephropathy except for extracellular fluid volume expansion. Pharmacological prophylaxis such as the administration of antioxidants, including N-acetylcysteine (NAC), theophylline, ascorbic acid, or statins, has been proposed, but none of these agents have shown a clear benefit or have been approved for clinical use [2]. Therefore, it is essential to understand the pathogenesis of contrast agent-induced nephropathy and to evaluate the scientific evidence to guide future research in this area.

*Artemisia argyi* is commonly known as mugwort, a herb used to treat inflammatory diseases [8], menstrual disorders [9], and dysmenorrhea [10] in traditional Asian medicine. Recently, various beneficial effects such as anticancer, antioxidant, immunomodulatory, anti-inflammatory, as well as neuroprotective effects have been documented in various studies. *A. argyi* is a source of bioactive compounds. Its main bioactive compounds are flavonoids, dimeric sesquiterpenoids, organic acids, phenolic compounds, and coumarins [11]. The antioxidant effect of eupatilin, a major flavonoid of *A. argyi*, was evaluated against hydrogen peroxide (H_2_O_2_)-induced oxidative damage in feline esophageal epithelial cells (EECs) [12]. Isoartemisolide, a primary sesquiterpenoid-monoterpenoid dimer of *A. argyi*, exhibits neuroprotective effects against lipopolysaccharide-activated BV-2 microglial cells [13]. Furthermore, 3,5-dicaffeoylquinic acid, a major organic acid, has also been reported to prevent trimethyltin-induced neuronal apoptosis in mice [14].

We previously demonstrated the protective effects of an extract of *A. argyi* against damage induced by cisplatin and iodixanol in a renal proximal tubular LLC-PK1 cells [15,16]. Eupatilin isolated from *A. argyi* has been identified as a flavonoid that protects LLC-PK1 cells from cisplatin-induced cell damage by ameliorating apoptosis [16]. Furthermore, some phenolic compounds, as well as flavonoids isolated from *A. argyi*, are more effective than NAC against iodixanol-induced damage in LLC-PK1 cells. Among them, artemetin, identified as a flavonoid, and methyl caffeate, identified as a phenolic compound, are responsible for the protective activities against cytotoxicity caused by iodixanol in LLC-PK1 cells [15,17]. Nevertheless, more research is required to understand the biological activities of *A. argyi* and its phytochemical components in the treatment of nephrotoxicity.

Thus, based on the merits of *A. argyi*, 9 sesquiterpene compounds were isolated and identified from *A. argyi*. We investigated the protective effects of these 9 sesquiterpenes against iodixanol-induced cytotoxicity in cultured renal tubular cells. This study was designed to examine whether this protective effect is mediated through antioxidant signaling pathways or anti-apoptotic signaling pathways.

## 2. Results

### 2.1. General Analytical Procedures

NMR spectra were determined using a Varian 500-MHz NMR spectrometer. Tetramethylsilane was used as an internal standard and chemical shifts were reported in ppm (*δ*). Column chromatography (CC) was conducted using silica gel (Kieselgel 60, 230–400 mesh, Merck, Darmstadt, Germany), and Sephadex LH-20 (18–111 µm, GE Healthcare AB, Stockholm, Sweden). MPLC (Isolera^TM^ One, Biotage AB, Uppsala, Sweden) silica gel CC was conducted using Biotage^®^ equipment with a Biotage^®^ SNAP cartridge HP-SIL (25–100 g) silica gel column. Preparative HPLC was conducted using the Varian Prostar 210 system with a YMC-Pack ODS-A column (5 μm, 250 × 20 mm i.d., YMC, Kyoto, Japan). ESI-MS data were recorded on an LCQ Fleet Ion Trap mass spectrometer (Thermo Scientific, Madison, WI, USA). 

### 2.2. Plant Material and Isolation of Sesquiterpene Compounds

The leaves of *Artemisia argyi* H. Lév. and Vaniot were obtained from Gyeongdong herbal medicine market (Seoul, South Korea) and a voucher specimen (accession number: AA1-103-130429) was stored at the Department of Biosystems and Biotechnology, Korea University, Seoul, Korea. The plant material was identified as reported previously [15].

The MeOH extract (420 g) was prepared form dried leaves of *A. argyi* (3 kg) as reported previously [15]. Sequentially, it was partitioned with *n*-hexane and EtOAc to afford *n*-hexane (KO1-103-1, 50 g) and EtOAc (KO1-103-2, 78 g) soluble extracts. The EtOAc extract (70 g) was applied to CC on silica gel and gradient elution with CHCl_3_–MeOH (1:0 to 1:1) to yield 9 fractions KO1-107-1 to -9. Fraction KO1-107-5 (25 g) was separated by silica gel CC gradient elution with CHCl_3_–acetone (1:0 to 1:1) to give 8 fractions KO1-120-1 to -8. Eupatilin (559.8 mg) was precipitated from fraction KO1-120-3, and the remainder of this fraction (4.14 g) was applied to CC on Sephadex LH-20 eluted with CHCl_3_–MeOH (1:1) to yield 11 fractions (KO1-122-1 to -11). KO1-122-5 (1.18 g) was further fractionated using MPLC silica gel CC eluted with CHCl_3_–MeOH (97:3) to yield KO1-123-1 to -8. The combined fraction KO1-123-1 to -3 (375 mg) was fractionated using MPLC silica gel CC gradient elution with *n*-hexane–EtOAc (1:0 to 1:1) to yield 7 fractions KO1-126-1 to -7. Fraction KO1-126-3 (66.3 mg) was separated by preparative HPLC (70–100% MeOH in H_2_O) to yield KO1-128-3 (17.7 mg), which was purified by preparative HPLC (70% acetonitrile in H_2_O) to afford moxartenolide (**1**, 7.0 mg) [18]. The purification of fraction KO1-126-5 (98.9 mg) was applied to a silica gel CC gradient elution with CHCl_3_–MeOH (1:0 to 1:1) resulting in the preparation of fractions KO1-132-1 to -6. Fraction KO1-132-3 (16.8 mg) was purified by preparative HPLC (70% MeOH in H_2_O) to afford dehydromatricarin A (**2**, 9.7 mg) [19]. Fraction KO1-120-5 (3.3 g) was separated on a column of reversed-phase C_18_ silica gel, gradient elution with MeOH–H_2_O (20–100%, MeOH in H_2_O), to yield 15 sub-fractions (TH5-23-1 to -15). TH5-23-4 (128.7 mg) was applied to silica gel CC gradient elution with *n*-hexane–EtOAc (1:0 to 0:1), resulting in the preparation of fractions TH5-41-1–TH5-41-5. Fraction TH5-41-1 (2.5 mg) was purified by preparative HPLC (60% MeOH in H_2_O) to yield tuberiferin (**3**, 3.0 mg) [20]. TH5-23-5 (113.0 mg) was subjected to silica gel CC gradient elution with *n*-hexane–EtOAc (1:0 to 0:1), resulting in the preparation of fractions TH5-27-1 to -6. Fraction TH5-27-4 was argyinolide G (**4**, 8.7 mg) **[21]**, and fraction TH5-27-3 (9.0 mg) was purified by preparative HPLC (40–60% MeOH in H_2_O) to yield deacetylmatricarin (**5**, 5.2 mg) [22]. TH5-23-7 (133.2 mg) was applied to silica gel CC gradient elution with *n*-hexane–EtOAc (1:0 to 0:1), leading to the isolation of rupicolin A (**6**, 4.2 mg) [23]. TH5-23-2 (223.8 mg) was subjected to silica gel CC gradient elution with CHCl_3_–MeOH (1–5%) resulting in the preparation of fractions TH5-31-1–TH5-31-5. Fraction TH5-31-2 (50 mg) was purified by preparative HPLC (40–60% MeOH in H_2_O) to yield acrifolide (**7**, 4.2 mg) [24]. Fraction TH5-31-4 (65 mg) was purified by preparative HPLC (40% MeOH in H_2_O) to yield 3-*epi*-iso-*seco*-tanapartholide (**8**, 7.7 mg) [25], and iso-*seco*-tanapartholide (**9**, 3.4 mg) [25].

### 2.3. Cell Culture

Porcine renal proximal tubular cells (LLC-PK1) were obtained from the American Type Culture Collection (Rockville, MD, USA) and grown in Dulbecco’s Modified Eagle Medium (Cellgro, Manassas, VA, USA), supplemented with 10% fetal bovine serum, 100 U/mL penicillin, 4 mM L-glutamine, and 100 μg/mL streptomycin (Invitrogen Co., Grand Island, NY, USA). Cells were incubated in an atmosphere of 5% CO_2_ at 37 °C.

### 2.4. Cellular Viability Test Using the Ez-Cytox Assay

Cell viability was determined using an Ez-Cytox assay kit (Daeil Lab service, Seoul, Korea). LLC-PK1 cells (1 × 10^4^ cells per well in 96-well culture plates). Cells were treated with the indicated concentrations of sesquiterpene compounds and incubated for 2 h, and then iodixanol (25 mg/mL) was treated to each well. After incubation for 3 h, 10 μL of the Ez-Cytox assay reagent was treated to each well. The absorbance values at 450 nm were assessed using a microplate reader for the calculation of cell viability. Ez-Cytox assay reagent contains water-soluble tetrazolium salt, which are reduced only in live cells that have mitochondrial activity. The percentage of cell viability was determined by calculating absorbance values of treated cells divided by the absorbance values of untreated cells, multiplied by 100%.

### 2.5. Quantification of ROS Levels Using DCF Staining

To assess ROS formation in LLC-PK1 cells, ROS levels were determined using DCFH-DA reagent (Sigma Aldrich, St. Louis, MO, USA). LLC-PK1 cells (4 × 10^5^ cells per well in 6-well plates) were incubated for 24 h for adhesion, and it was treated with the indicated concentrations of sesquiterpene compounds and incubated for 2 h, and then iodixanol (25 mg/mL) was treated to each well. After incubation for a further 3 h, 10 µM DCFH-DA was treated to each well and incubated for 30 min in the dark. After washing with PBS, the fluorescence intensity of DCF was measured at 495 (excitation wavelength) and 517 nm (emission wavelength) using a SPARK 10M fluorescence microplate reader (Tecan, Männedorf, Switzerland).

### 2.6. Evaluation of Apoptosis

Apoptotic cell death was evaluated using a Tali Apoptosis Assay Kit (Invitrogen, Temecula, CA, USA). This kit enables the identification of apoptotic cells stained with annexin V Alexa Fluor 488 and the discrimination of apoptotic cells from necrotic cells stained with both propidium iodide (PI) and annexin V Alexa Fluor 488. LLC-PK1 cells (4 × 10^5^ cells per well in 6-well culture plates) were incubated for 24 h for adhesion. Cells were then treated with the indicated concentrations of sesquiterpene compounds and incubated for 2 h, and then iodixanol (25 mg/mL) was treated to each well. After incubation for a further 3 h, the cells were harvested and the pellets were re-suspended in binding buffer (5 × 10^5^ cell/100 μL) and incubated with 5 μL of annexin V Alexa Fluor 488 and 1 μL of PI for 30 min in the dark at room temperature. The percentages of apoptotic, necrotic, and live cells in the cell population were automatically calculated using a Tali image-based cytometer (Invitrogen, Temecula, CA, USA).

### 2.7. Immunofluorescence Staining

LLC-PK1 cells (3 × 10^4^ cells per well in 8-well chamber slides) were incubated for 24 h for adhesion. Cells were then treated with the indicated concentrations of sesquiterpene compounds and incubated for 2 h, and then iodixanol (25 mg/mL) was treated to each well. After incubation for 3 h, cells were fixed with paraformaldehyde for 30 min at room temperature and then permeabilized with 0.1% Triton X-100 in PBS. The cells were probed with Nrf2 primary antibody (# 14596S, clone D9J1B) purchased from Cell Signaling Technology (Danvers, MA, USA) at 4 °C overnight in the dark. After being washed with 0.01% PBS-T, the cells were probed with FITC-conjugated goat anti-rabbit IgG secondary antibody (Cell Signaling Technology) at 4 °C for overnight in the dark. After being washed with 0.01% PBS-T, the slides were mounted with ProLong^®^ Gold antifade reagent (Thermo Fisher Scientific, Waltham, MA, USA) containing DAPI-stained nuclei. Stained cells were photographed using a fluorescence microscope (IX 50) equipped with a CCD camera.

### 2.8. Western Blotting Analysis

LLC-PK1 cells (4 × 10^5^ cells per well in 6-well plates) were incubated for 24 h for adhesion. Cells were treated with the indicated concentrations of sesquiterpene compounds and incubated for 2 h, and then iodixanol (25 mg/mL) was treated to each well. After incubation for further 3 h, to extract whole protein, the collected pellets were lysed using RIPA buffer (Cell Signaling Technology) supplemented with 1 mM phenylmethylsulfonyl fluoride and 1× EDTA-free protease inhibitor cocktail for 20 min at 4 °C. To extract nuclear protein and cytoplasmic protein, the collected pellets were lysed using cytoplasmic extraction buffer (10 mM HEPES, pH 7.9, 10 mM KCl, 0.1 mM EDTA, 0.1% NP-40) supplemented with 1× EDTA-free protease inhibitor cocktail for 15 min at 4 °C. Following centrifugation, the supernatant containing the cytoplasmic protein was collected. The remaining pellet was resuspended in RIPA buffer supplemented with 1 mM PMSF and 1× EDTA-free protease inhibitor cocktail for 15 min at 4 °C. Following centrifugation, the supernatant containing the nuclear protein was collected. Equal amounts of protein sample were separated using electrophoresis as reported previously [15]. Then, the membrane was incubated with primary antibodies against Nrf2 (# 12721S, 1:1000), HO-1 (# 43966S, 1:1000), Lamin B1 (# 13435S, 1:1000), P-JNK(# 4668S, 1:1000), JNK(# 9252S, 1:1000), P-ERK(# 4376S, 1:1000), ERK (# 9102S, 1:1000), P-P38 (# 4511S, 1:1000), P38(# 8690S, 1:1000), Bax (# 2772S, 1:1000), Bcl-2 (# 3498S, 1:1000), cleaved caspase-8 (# 8592S, 1:1000), cleaved caspase-9 (# 20750S, 1:1000), cleaved caspase-3 (# 9661S, 1:1000), and glyceraldehyde 3-phosphate dehydrogenase (GAPDH) (# 2118S, 1:1000) for 1 h at room temperature. Then the membrane was sequentially incubated with horseradish peroxidase-conjugated rabbit secondary antibody for 1 h at room temperature. all antibodies were purchased from Cell Signaling Technology. Enhanced chemiluminescence (ECL) Advance Western Blotting Detection Reagents (GE Healthcare; Cambridge, UK) was used to develop protein bands. The protein bands were visualized using a FUSION Solo Chemiluminescence System (PEQLAB Biotechnologie GmbH; Erlangen, Germany).

### 2.9. Statistical Analysis

All assays were performed with triplicate samples and were repeated at least three times. Statistical analyses were assessed using analysis of variance followed by a multiple comparison test with a Bonferroni adjustment. The analysis was performed using SPSS ver. 19.0 (SPSS Inc., Chicago, Il, USA). *P*-values less than 0.05 were considered statistically significant.

## 3. Results

### 3.1. Identification of Compounds 1–9

**Figure 1:**
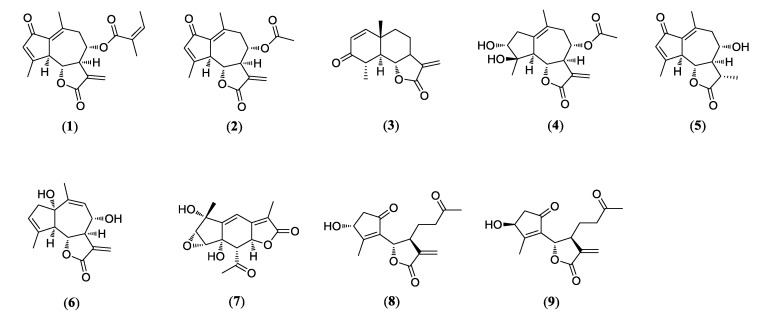
Chemical structures of nine sesquiterpenes including moxartenolide (compound **1**), dehydromatricarin A (compound **2**), tuberiferin (compound **3**), argyinolide G (compound **4**), deacetylmatricarin (compound **5**), rupicolin A (compound **6**), acrifolide (compound **7**), 3-*epi*-iso-*seco*-tanapartholide (compound **8**), and iso-*seco*-tanapartholide (compound **9**) isolated from *Artemisia argyi*.

Nine compounds (**1**–**9**) were isolated from the Artemisia argyi in this study. The purity of the isolates (>95%) was determined by NMR. The chemical structures of compounds **1**–**9** were identified as moxartenolide (compound **1**) [18], dehydromatricarin A (compound **2**) [19], tuberiferin (compound **3**) **[20]**, argyinolide G (compound **4**) **[21]**, deacetylmatricarin (compound **5**) **[22]**, rupicolin A (compound **6**) [23], acrifolide (compound **7**) **[24]**, 3-epi-iso-seco-tanapartholide (compound **8**) [25], iso-seco-tanapartholide (compound **9**) **[25]**, by 1D and 2D NMR spectroscopic data and by comparison with published values (Figure 1).

### 3.2. Comparison of the Protective Effects of 9 Sesquiterpenes Isolated from A. argyi on Contrast Agent-Induced Cytotoxicity in LLC-PK1 Cells 

**Figure 2:**
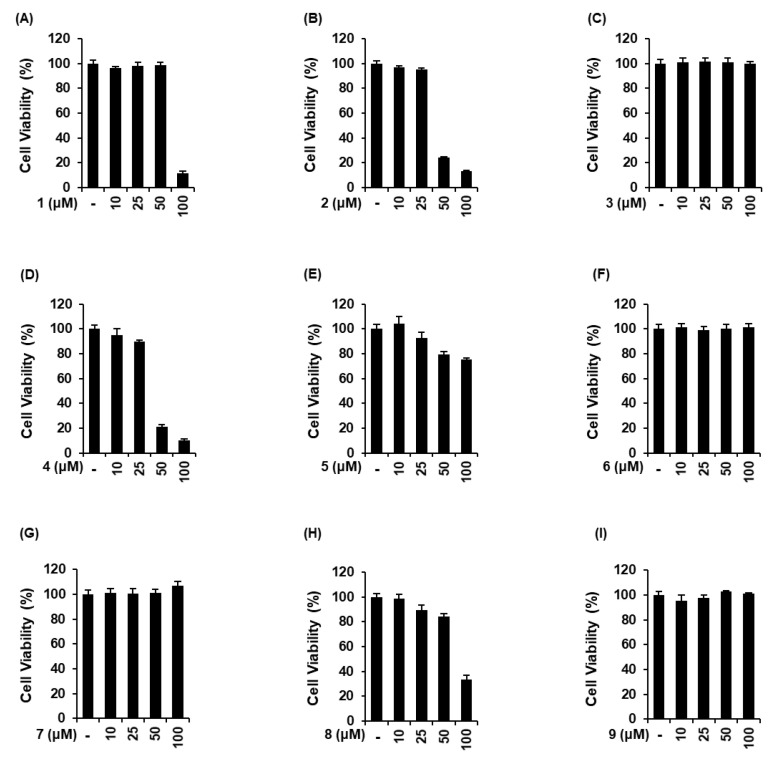
Comparison in the effects of 9 sesquiterpenes isolated from *Artemisia argyi* in LLC-PK1 cells. (**A**–**I**) Cells were exposed to nine sesquiterpene compounds including (**A**) moxartenolide (compound **1**), (**B**) dehydromatricarin A (compound **2**), (**C**) tuberiferin (compound **3**), (**D**) argyinolide G (compound **4**), (E) deacetylmatricarin (compound **5**), (**F**) rupicolin A (compound **6**), (**G**) acrifolide (compound **7**), (**H**) 3-*epi*-iso-*seco*-tanapartholide (compound **8**), and (**I**) iso-*seco*-tanapartholide (compound **9**), and cell viability was measured using an Ez-Cytox cell viability assay kit (mean ± SD, *n* = 3, * *p* < 0.05 compared with untreated LLC-PK1 cells).

The non-toxic dose of 9 sesquiterpenes isolated from *A. argyi* was determined using a cell viability assay on LLC-PK1 cells. Moxartenolide (compound **1**), dehydromatricarin A (compound **2**), argyinolide G (compound **4**), deacetylmatricarin (compound **5**), and 3-*epi*-iso-*seco*-tanapartholide (compound **8**) were cytotoxic at concentrations of 25 μM and above (Figure 2A,B,D,E,H). However, tuberiferin (compound **3**), rupicolin A (compound **6**), acrifolide (compound **7**), and iso-*seco*-tanapartholide (compound **9**), did not show any toxic effect at 10, 25, 50, and 10 μM (Figure 2C,F,G,I), respectively. Nine sesquiterpenes at concentrations of 10 μM and below were used to evaluate the protective effects of 9 sesquiterpenes isolated from *A. argyi* on iodixanol-induced cytotoxicity in LLC-PK1 cells. 

**Figure 3:**
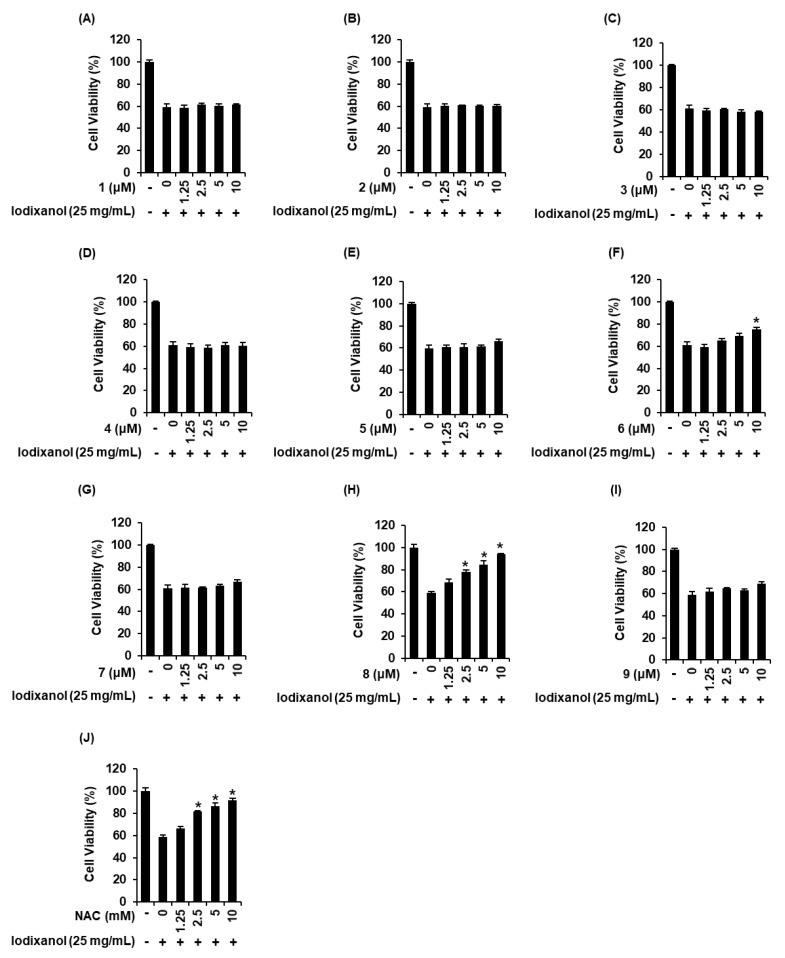
Comparison in the protective effects of nine sesquiterpenes isolated from *Artemisia argyi* on iodixanol-induced cytotoxicity in LLC-PK1 cells. (**A**–**J**) Cells were exposed to 25 mg/mL iodixanol in the presence or absence of nine sesquiterpene compounds including (**A**) moxartenolide (compound **1**), (**B**) dehydromatricarin A (compound **2**), (**C**) tuberiferin (compound **3**), (**D**) argyinolide G (compound **4**), (**E**) deacetylmatricarin (compound **5**), (**F**) rupicolin A (compound **6**), (**G**) acrifolide (compound **7**), (**H**) 3-*epi*-iso-*seco*-tanapartholide (compound **8**), and (**I**) iso-*seco*-tanapartholide (compound **9**) and (**J**) N-acetyl cysteine (NAC) and cell viability was measured using an Ez-Cytox cell viability assay kit (mean ± SD, *n* = 3, * *p* < 0.05 compared with iodixanol-treated LLC-PK1 cells).

We compared the protective effects of 9 sesquiterpenes isolated from *A. argyi* on iodixanol-induced cytotoxicity in LLC-PK1 cells. As shown in Figure 3, 25 mg/mL iodixanol significantly decreased cell viability by approximately 40% compared with non-treated cells (100%). As shown in Figure 3F, compound **6** had the protective effect at 10 μM, with a cell survival rate of 75.1% ± 1.9%. As shown in Figure 3H, at concentrations of 2.5, 5, and 10 μM of compound **8**, LLC-PK1 cell viability was 77.7% ± 2.2%, 84.5% ± 3.2%, and 93.9% ± 0.6% compared with iodixanol-treated cells. NAC had a similar protective effect to compound **8** at a concentration 1000 times higher than the concentration of compound **8**, with a cell survival rate of 91.6% ± 1.9% (Figure 3J). Other compounds exhibited no protective effects at any of the concentrations (Figure 3A–E,G,I). Subsequently, mechanistic studies were performed using compound **8** because it was shown that treatment with this compound appeared to be sufficiently protective on iodixanol-induced cytotoxicity in LLC-PK1 cells.

### 3.3. Effect of 3-Epi-Iso-Seco-Tanapartholide on Contrast Agent-Induced Morphological Changes and ROS Generation in LLC-PK1 Cells

**Figure 4:**
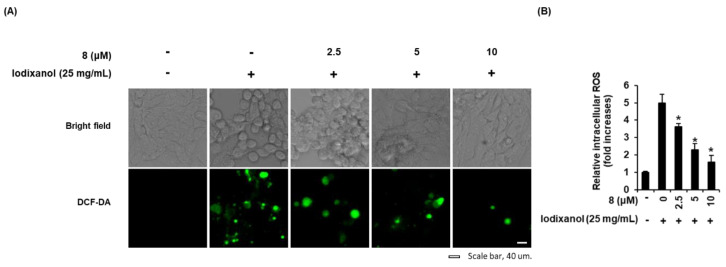
Effects of 3-*epi*-iso-*seco*-tanapartholide (compound **8**) isolated from *Artemisia argyi* on iodixanol-induced morphological changes and ROS generation in LLC-PK1 cells. (**A**) Cells were exposed to 25 mg/mL of iodixanol in the absence or presence of compound **8** and stained with H_2_DCFDA. The fold-increases of intracellular ROS levels detected by DCF fluorescence (green fluorescence) are represented as a bar graph (mean ± SD, *n* = 3, * *p* < 0.05 compared with iodixanol-treated LLC-PK1 cells). (**B**) Representative bright field and fluorescence images were obtained using an IX50 fluorescence microscope. White scale bar, 40 µm.

The effects of compound **8** on morphological changes and ROS generation were determined in LLC-PK1 cells exposed to 25 mg/mL iodixanol in the absence or presence of compound **8** using DCF staining. As shown in the cell images obtained using IX50 fluorescence microscopy (Figure 4A), untreated cells had typical healthy morphology while cells treated with 25 mg/mL iodixanol were flattened or rounded and showed loss of adhesion. These morphological changes were decreased by pretreatment with 2.5, 5, and 10 μM of compound **8**. At the same time, ROS generation was also visualized by IX50 fluorescence microscopy (Figure 3D). The observed green fluorescence intensity of DCF (fold increase of ROS) was increased significantly by 4.9 ± 0.4-fold after treatment with 25 mg/mL iodixanol, whereas it was decreased by 3.6 ± 0.1-, 2.2 ± 0.3-, and 1.5 ± 0.3-fold by pretreatment with 2.5, 5, and 10 μM of compound **8** prior to treatment with iodixanol (Figure 4A,B).

### 3.4. Effects of 3-Epi-Iso-Seco-Tanapartholide on the Expression Levels of Nrf2/HO-1 Proteins and Nuclear Translocation of Nrf2 Associated with Antioxidant Pathways in Iodixanol-Treated LLC-PK1 Cells

**Figure 5:**
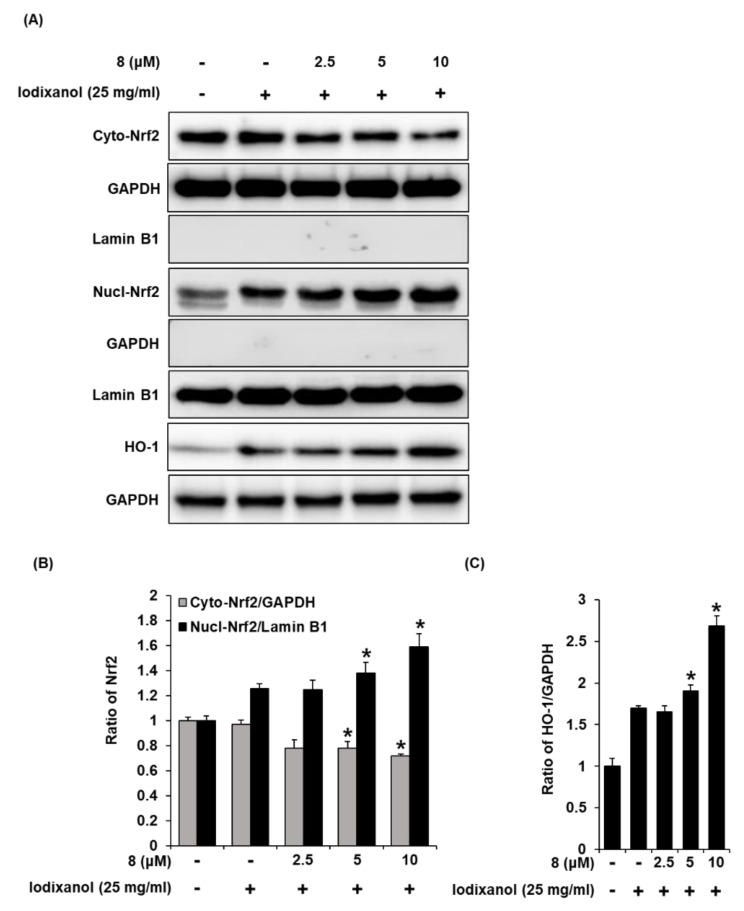
The effect of 3-*epi*-iso-*seco*-tanapartholide (compound **8**) on the nuclear expression of Nrf2 and HO-1 proteins associated with antioxidant pathways in LLC-PK1 cells exposed to iodixanol. (**A**) LLC-PK1 cells were treated with 25 mg/mL iodixanol in the absence or presence of **8**, and western blot analysis was conducted using antibodies for Nrf2, Lamin B1, HO-1, and glyceraldehyde 3-phosphate dehydrogenase (GAPDH). Lamin B1 and GAPDH were used as a cytosolic and nuclear controls for western blot analysis, respectively. (**B**) and (**C**) The ratios of nuclear Nrf2, cytosolic Nrf2, and HO-1 compared with untreated cells are represented as a bar graph (mean ± SD, *n* = 3, * *p* < 0.05 compared with iodixanol-treated LLC-PK1 cells).

**Figure 6:**
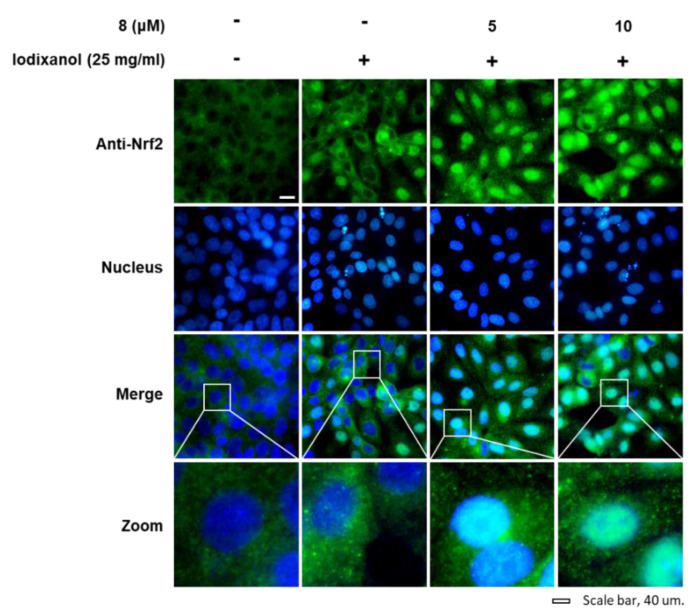
The effects of 3-*epi*-iso-*seco*-tanapartholide (compound **8**) on the nuclear translocation of Nrf2 in LLC-PK1 cells exposed to iodixanol. LLC-PK1 cells were treated with 25 mg/mL iodixanol in the presence or absence of **8**, and immunofluorescence staining was performed using antibodies for Nrf2 that were visualized with a goat anti-rabbit IgG-heavy- and light-chain FITC-conjugated antibody (green fluorescence) and mounted with ProLong^®^ Gold antifade reagent with DAPI-stained nuclei (DAPI). The white arrows indicate the presence of Nrf2. White scale bar, 40 µm.

The effects of compound **8** on translocation of Nrf2 to the nucleus and HO-1 expression associated with antioxidant pathways were determined in LLC-PK1 cells exposed to 25 mg/mL iodixanol in the absence or presence of compound **8** using western blotting and immunofluorescence staining. As shown in Figure 5A, while treatment with 25 mg/mL iodixanol increased the nuclear translocation of Nrf2 compared with non-treated cells, pretreatment with compound **8** at 5 and 10 μM prior to treatment with iodixanol further enhanced this effect. In addition, compound **8** increased HO-1 protein expression. As shown in Figure 6, images of immunofluorescence staining (green fluorescence) for Nrf2 along with DAPI (blue fluorescence) as a nuclear counterstain showed a similar finding. In the non-treated cells, Nrf2 green fluorescence was primarily found in the cytoplasm. While treatment with 25 mg/mL iodixanol increased the intensity of Nrf2 green fluorescence in nuclei compared with non-treated cells, pretreatment with compound **8** at 5 and 10 μM prior to treatment with iodixanol further enhanced this effect, indicating the promotion of Nrf2 nuclear translocation.

### 3.5. Effect of 3-Epi-Iso-Seco-Tanapartholide on Iodixanol-Induced Apoptosis in LLC-PK1 Cells

**Figure 7:**
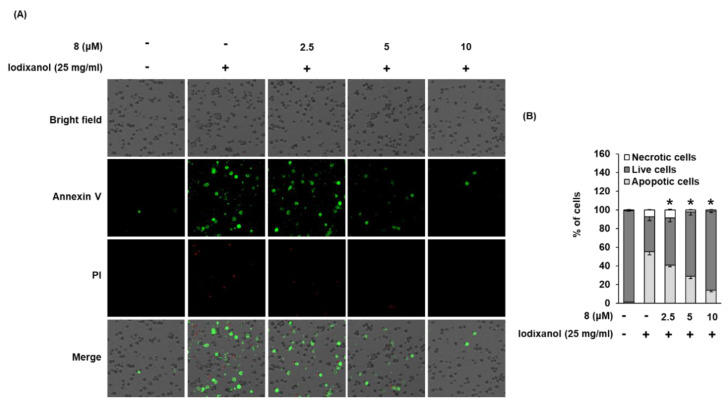
Effect of 3-*epi*-iso-*seco*-tanapartholide (compound **8**) isolated from *Artemisia argyi* on iodixanol-induced apoptosis in LLC-PK1 cells. (**A**) Cells were treated to 25 mg/mL of iodixanol in the presence or absence of compound 8 for 24 h, and stained with annexin V Alexa Fluor 488 (green fluorescence) and PI (red fluorescence). Images were quantitatively determined using a Tali image-based cytometer. (**B**) The percentages of apoptotic, necrotic, and live cells are represented as a bar graph (mean ± SD, *n* = 3, * *p* < 0.05 compared with iodixanol-treated LLC-PK1 cells).

Staining with annexin V Alexa Fluor 488, an apoptosis indicator, and propidium iodide (PI), a necrosis indicator, was used to assess the effect of compound **8** on iodixanol-induced apoptosis in LLC-PK1 cells. As shown in Figure 7B, the percentage of cells stained with annexin V Alexa Fluor 488 (green fluorescence) undergoing apoptosis increased significantly by 54.9% ± 3.2% after treatment with 25 mg/mL iodixanol, whereas it was decreased by treatment with 2.5, 5, and 10 μM of compound **8**, prior to treatment with iodixanol, to 40.6% ± 1.5%, 28.8% ± 2.8%, and 13.7% ± 1.7%, respectively. However, the percentage of cells stained with PI (red fluorescence), indicating necrosis, did not show significant changes.

### 3.6. Effect of 3-Epi-Iso-Seco-Tanapartholide on Expression Levels of Bcl-2, Bax, Cleaved Caspase-8, -9, and -3 Proteins Associated with Apoptosis Pathways in Iodixanol-Treated LLC-PK1 Cells

**Figure 8:**
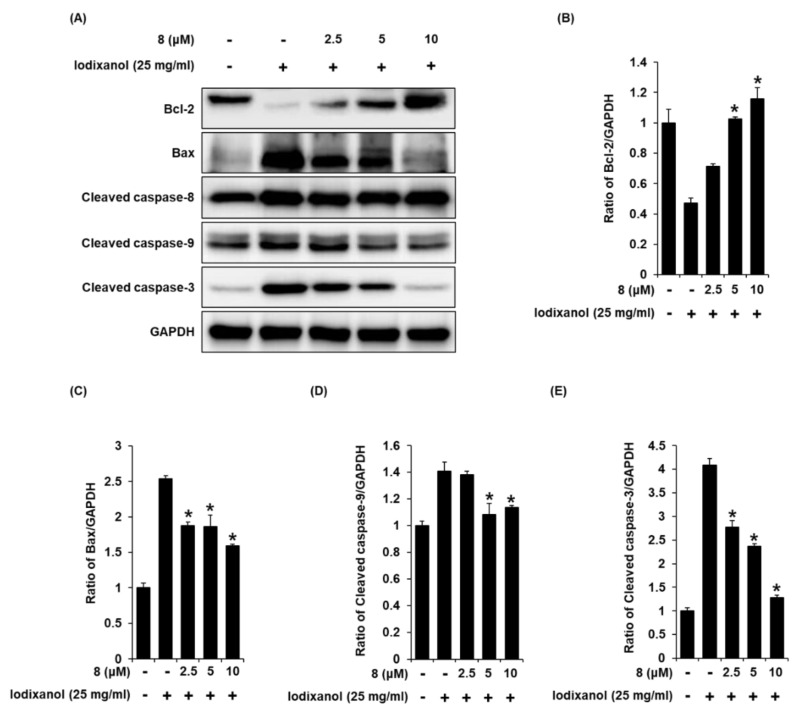
The effect of 3-*epi*-iso-*seco*-tanapartholide (compound **8**) on the expression of Bcl-2, Bax, cleaved caspase-8, -9, and -3 proteins associated with apoptosis pathways in LLC-PK1 cells exposed to iodixanol. (**A**) LLC-PK1 cells were treated with 25 mg/mL iodixanol in the absence or presence of compound **8**, and western blot analysis was performed using antibodies for Bax, Bcl-2, cleaved caspase-8, -9, -3, and GAPDH. (**B**–**E**) The ratios of Bcl-2, Bax, cleaved caspase-9, and -3 compared with the untreated cells are represented as a bar graph (mean ± SD, *n* = 3, * *p* <0.05 compared with iodixanol-treated LLC-PK1 cells).

To determine the effects of compound **8** on the expression levels of proteins associated with apoptosis pathways in iodixanol-treated LLC-PK1 cells, the expression levels of Bax, Bcl-2, cleaved caspase-8, -9, and -3 proteins in LLC-PK1 cells exposed to 25 mg/mL iodixanol in the absence or presence of compound **8** were detected using western blotting. As shown in Figure 8A, compared with the non-treated cells, treatment with 25 mg/mL iodixanol significantly increased expression levels of Bax, cleaved caspase-3, and cleaved caspase-9, whereas it significantly decreased the expression level of Bcl-2. However, pretreatment with compound **8** at 10 μM enhanced Bcl-2 expression levels and inhibited the expression of Bax, cleaved caspase-9, and caspase-3. However, pretreatment with compound **8** did not change the expression level of cleaved caspase-8.

### 3.7. Effect of 3-Epi-Iso-Seco-Tanapartholide on the Protein Expressions of JNK, ERK, and p38 Proteins Associated with MAPK Pathways in Iodixanol-Treated LLC-PK1 Cells

**Figure 9:**
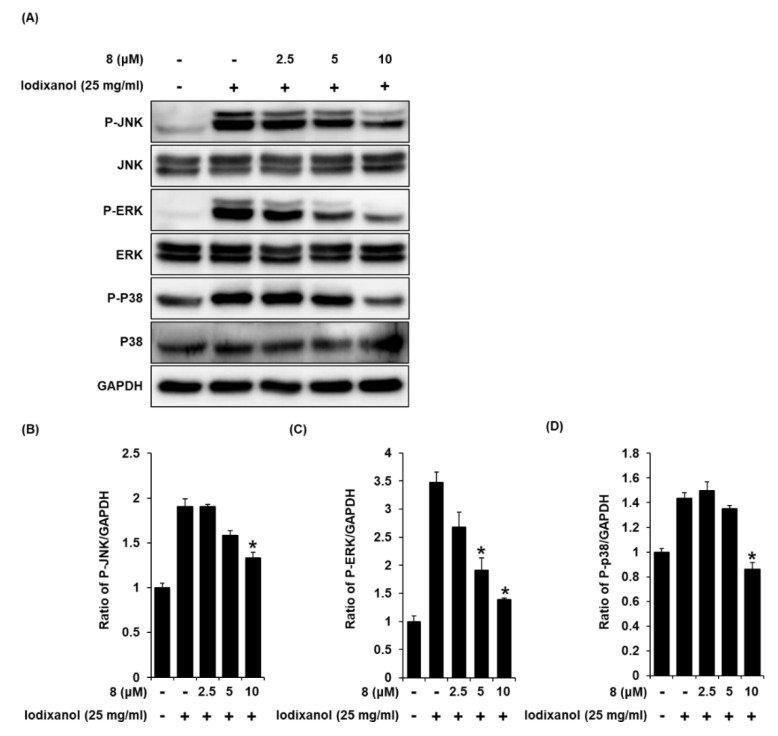
The effects of 3-*epi*-iso-*seco*-tanapartholide (compound **8**) on the expression of JNK, ERK, p38 proteins associated with MAPK pathways in LLC-PK1 cells treated with iodixanol. (**A**) LLC-PK1 cells were treated with 25 mg/mL iodixanol in the absence or presence of compound 8, and western blot analysis was performed using antibodies for P-JNK, JNK, P-ERK, ERK, P-P38, P38, and GAPDH. (**B**–**D**) The ratios of JNK, ERK, and P38 phosphorylation compared with untreated cells are represented as a bar graph (mean ± SD, *n* = 3, * *p* < 0.05 compared with iodixanol-treated LLC-PK1 cells).

To detect the expression of JNK, ERK, and p38 proteins associated with MAPK pathways in LLC-PK1 cells treated to 25 mg/mL iodixanol in the presence or absence of compound **8**, western blot analysis was used. As shown in Figure 9A, compared with non-treated cells, treatment with 25 mg/mL iodixanol significantly increased JNK, ERK, and p38 expression. However, pretreatment with compound **8** at 10 μM inhibited JNK, ERK, and p38 expression.

### 3.8. Effects of Combined Treatment with Inhibitors of MAPK Pathways (SB203580 and U0126) and 3-Epi-Iso-Seco-Tanapartholide on Iodixanol-Treated LLC-PK1 Cells

**Figure 10:**
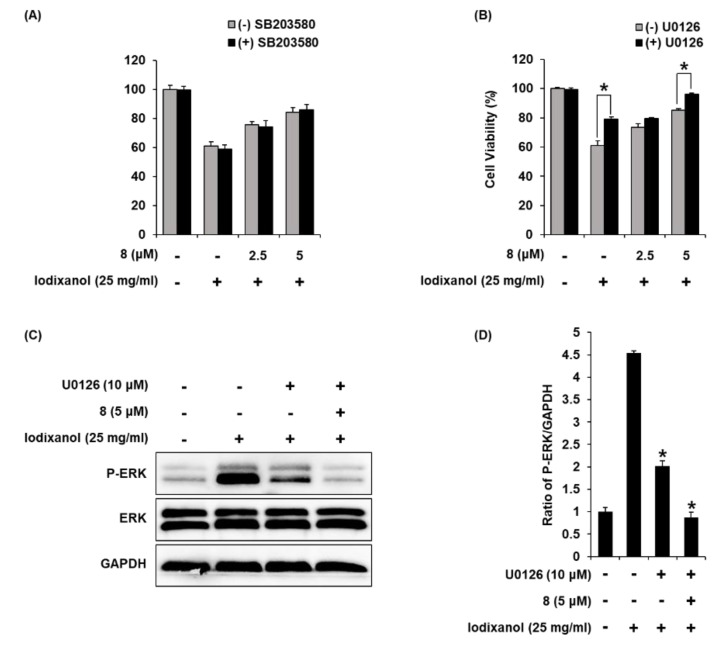
The effects of U0126 (ERK inhibitor) on the protective effects of 3-*epi*-iso-*seco*-tanapartholide (compound **8**) in iodixanol-induced cytotoxicity in LLC-PK1 cells. (**A**) and (**B**) Cells were treated with 25 mg/mL iodixanol and/or (**A**) SB203580 (P38 inhibitor, 15 μM) or (**B**) U0126 (10 μM) after pre-treatment with compound **8** (2.5 and 5 μM) and cell viability was measured using an Ez-Cytox cell viability assay kit (mean ± SD, *n* = 3, * *p* < 0.05 for differences between the indicated pair of treatments. (**C**) LLC-PK1 cells were treated with 25 mg/mL iodixanol and/or U0126 (10 μM) after pre-treatment with compound **8** (5 μM), and western blot analysis was performed using antibodies for P-ERK, ERK, and GAPDH. (**D**) The ratios of ERK phosphorylation compared with untreated cells are represented as a bar graph (mean ± SD, *n* = 3, * *p* < 0.05 compared with iodixanol-treated LLC-PK1 cells).

To elucidate the involvement of P38 and ERK proteins, we investigate the effects of combined treatment with compound **8** and inhibitors of P38 and ERK proteins on iodixanol-induced LLC-PK1 cell death. LLC-PK1 cells were treated with 25 mg/mL iodixanol and/or 15 μM of SB203580 (p38 inhibitor) or 10 μM of U0126 (ERK inhibitor) after pre-treatment with 2.5 μM and 5 μM compound **8**. The effects of combined treatment were determined using a cell viability assay on LLC-PK1 cells. As shown in Figure 10A, when 15 μM of SB203580 was treated alone, there was no toxicity to the cells. Iodixanol significantly decreased cell viability by approximately 40% compared with non-treated cells (100%), but SB203580 showed no protective effect. The protective effects of 2.5 and 5 μM of compound **8** correlated with earlier results (Figure 3H), increasing the cell viability to 73.33% ± 0.49% and 85.01% ± 0.81%. 

As shown in Figure 10B, when 10 μM of U0126 was treated alone, there was no toxicity to the cells. In addition, the reduced cell viability by approximately 40% after treatment with iodixanol was significantly increased to 79.62% ± 2.72% after treatment with U0126. At concentrations of 2.5 and 5 μM of compound **8**, cell viability was 75.58% ± 2.07% and 84.35% ± 3.06% compared with iodixanol-treated cells, consistent with earlier results (Figure 3H). Moreover, combined treatment with U0126 and compound **8** showed synergistic protective effects. The cell viability was increased to 96.05% ± 0.97% after combined treatment with 10 μM of U0126 and 5 μM of compound **8**. To elucidate the involvement of ERK protein, we investigated the effects of combined treatment with U0126 and compound **8** on cisplatin-induced LLC-PK1 cells treated with 25 mg/mL iodixanol and/or 10 μM of U0126 after pre-treatment with 5 μM of compound **8**, western blot analysis was used. As shown in Figure 10C,D, compared with untreated cells, treatment with 25 mg/mL iodixanol significantly increased ERK expression. However, pretreatment with U0126 at 10 μM inhibited ERK expression. Moreover, combined treatment with 10 μM of U0126 and 5 μM of compound **8** showed synergistic inhibitory effects.

## 4. Discussion

In this study, we examined the effect of nine sesquiterpenes isolated from *A. argyi* against iodixanol-induced cell damage, and the mechanism underlying its anti-apoptosis and antioxidant effects in LLC-PK1 cells. As a comparative study, the protective effects of 9 sesquiterpenes against iodixanol-induced cell damage caused by iodixanol in LLC-PK1 cells were evaluated. Iodixanol-induced cytotoxicity may affect changes in cellular energy metabolism and renal epithelial cell monolayers [26,27]. Interestingly, iso-*seco*-tanapartholide (compound **9**) exhibited no protective effect on iodixanol-induced LLC-PK1 cell death, whereas the approximately 60% survival of LLC-PK1 cells after treatment with iodixanol was increased to over 80% after pretreatment with 3-epimer of compound **9**, compound **8** at the nontoxic concentration. These protective effects against the direct cytotoxicity of iodixanol in LLC-PK1 cells might contribute to the mitigation of renal tubular injury. In addition, treatment of LLC-PK1 cells with iodixanol transformed the cells to being flattened and rounded and induced the loss of cell adhesion. This result suggested that iodixanol induced anchorage loss-dependent cell death, whereas pretreatment of cells with compound **8** prevented these morphological changes. Interestingly, compound **8** showed a similar effect at 1000 times lower concentrations than the concentration of NAC used as a positive control. Several studies have reported the efficacy of pharmacological prophylaxis of NAC, a known antioxidant, against contrast-induced nephropathy [28,29,30]. Studies in vivo show similar evidence to support the efficacy of antioxidants in preventing contrast-induced nephropathy [31,32,33]. These pharmacologic measures suggest that oxidative stress is a crucial mediator of contrast-induced nephropathy. Thus, compound **8** at 5 and 10 μM was used to evaluate its antioxidant effects in LLC-PK1 cells. 

It has been reported that the difference in antioxidant activity depends on the extraction solvents and the plant parts of *A. argyi*. Compare to H_2_O and EtOH leaf extracts, MeOH leaf extracts exhibit the highest antioxidant effect [34]. In addition, many of the sesquiterpene biological activities have been reported to be based on the antioxidant effect [35]. However, to the best of our knowledge, this is the first report on biological activity based on antioxidant effect of compound **8.** To determine the antioxidant effects of compound **8**, intracellular ROS levels were detected using DCF fluorescence (green fluorescence), which is produced by ROS-induced oxidation of DCFH-DA into DCF [36]. The fold-increases of emitted fluorescence were proportional to the intracellular ROS levels induced by iodixanol. The increased ROS concentrations were decreased after pretreatment with compound **8.** This result indicated that iodixanol induce oxidative stress, it accelerates ROS production, whereas it is reduced by ROS scavenging with compound **8**. In addition, considering the results of cell protection and antioxidant effect together, cell damage could be attenuated by ROS scavenging with compound **8**. 

The transcription factor Nrf2 regulates the antioxidant defense system, inducing transcription of antioxidant genes [37]. HO-1 is a rate-limiting enzyme of heme degradation, and its expression is regulated by Nrf2 in response to oxidative stress to protect the cells [38]. A previous study demonstrated that salvianolic acid B, identified as a phenolic acid derived from the roots of *Salvia miltiorrhiza*, attenuates oxidative stress as well as apoptosis after exposure to contrast agents in rats and HK-2 human proximal tubule cells. The underlying mechanism of these effects is the activation of the Nrf2/HO-1 pathway [39]. Similar observations were reported in our study. The results of western blotting and immunofluorescence staining showed that pretreatment with compound **8** prior to treatment with iodixanol caused the translocation of Nrf2 from the nucleus to the cytoplasm. This effect correlated with increased HO-1 expression. Thus, these results suggested that iodixanol-induced cytotoxicity was attenuated by eliminating iodixanol-induced oxidative stress through the induction of Nrf2-mediated HO-1 expression in LLC-PK1 cells after pretreatment with compound **8**, providing evidence in support of their action of antioxidant defense systems against the damaging effects of iodixanol-induced oxidative stress.

To determine if reduced cell viability after treatment with iodixanol is due to apoptosis or necrosis, and to measure the protective effect of compound **8**, images of cells stained with annexin V Alexa Fluor 488, PI, or the combination were quantitatively analyzed. Quantitative analysis showed that pretreatment with compound **8** decreased the percentage of apoptotic cells as indicated by green fluorescence, compared with iodixanol-treated LLC-PK1 cells. These results indicate the protective effects of compound **8** against iodixanol-induced apoptotic cell death. A previous study demonstrated that contrast agents induce apoptosis through the activation of caspase-9 and caspase-3 and increased ratio of Bax to Bcl-2 expression (Bax/Bcl-2) in LLC-PK1 cells [40,41,42]. In another study using the same cell line, treatment with the contrast agent induced apoptosis through both mitochondrial and caspase-9 (intrinsic) pathways and the death receptor/caspase-8 (extrinsic) pathway [43]. Similar results were described in our study. Compound **8** attenuated apoptosis by decreasing the expression levels of Bax, cleaved caspase-3, and cleaved caspase-9 and increasing the expression level of Bcl-2, without any change in the expression levels of cleaved caspase-8. These results suggested that compound **8** inhibits the intrinsic apoptosis pathway in iodixanol-treated LLC-PK1 cells. 

Three major subfamilies of MAPKs, including JNKs, ERKs, and p38 kinases are related to both oxidative stress and apoptosis [44]. The activation of p38 kinases induced by oxidative stress has been involved in the induction of HO-1 via Nrf2 [38]. In addition, ROS can lead to apoptosis through oxidative DNA damage in renal tubular epithelial cells in contrast-induced nephropathy [31]. 

In our study, pretreatment with compound 8 inhibited the phosphorylation of ERK, JNK, and p38 induced by iodixanol. Studies have reported that JNK and p38 induced by oxidative stress regulate apoptosis against contrast-induced nephropathy in vitro and in vivo [44,45]. In contrast, the role of ERK remains less clear than that of JNK and p38 and remains controversial, with conflicting evidence. There is evidence of similar roles of JNK and p38 and vice versa. Contrary to our findings, a previous study provides evidence for a protective role of ERK against contrast-induced nephropathy in vivo [45]. In the present study, treatment with U0126 attenuates iodixanol-induced LLC-PK1 cell death and this protective effect was synergistic when treated with 5 μM of compound **8**. Effect of combined treatment with U0126 and compound **8** was better than that of treatment with 5 μM of compound **8** alone. Furthermore, the combined treatment with U0126 and compound **8** showed synergistic inhibitory effects on phosphorylation of ERK. Thus inhibition of ERK might participate in a positive feedback abolishing iodixanol-induced LLC-PK1 cell death and enhancement of protective effect of 5 μM of compound **8.** Taken together, these results reiterated the protective effect of compound **8** in attenuating iodixanol-induced LLC-PK1 cell death that could be mediated at least in part by mechanisms linked to the antioxidant pathways, anti-apoptosis pathways, and MAPK pathway.

## 5. Conclusions

In this study, nine sesquiterpene compounds were isolated and identified from *A. argyi*. These sesquiterpenes protected LLC-PK1 cells from iodixanol-induced cytotoxicity. Compound **8**, with the most robust protection in the cell viability, inhibited oxidative stress through the induction of Nrf2 nuclear translocation and Nrf2-mediated HO-1 expression. It also inhibited apoptosis by decreasing the expression levels of Bax, cleaved caspase-8, -9, and -3 proteins and increasing the expression of Bcl-2 in the intrinsic mitochondrial apoptotic pathway. These effects might be partly mediated through phosphorylation of ERK. Although further in vivo studies of the protective effects of compound **8** against contrast agent-induced cell damage are required, our results provide basic scientific evidence that will help understand the protective mechanisms of compound **8** against contrast-induced cell damage.
